# Restorative initiatives: emerging insights from design, implementation and collaboration in five countries

**DOI:** 10.3389/frhs.2025.1472738

**Published:** 2025-02-28

**Authors:** Jo Wailling, Graham Cameron, Iwona Stolarek, Stephanie Turner, Beelah Bleakley, Nick O’Connor, Catriona Harwood, Michael Power, Kathryn Turner, Allison Kooijman, Nelly D. Oelke, David Gustafson, Rob Robson, Murray Anderson Wallace, Gerard Drennan, Jo Hughes, Jane K. O'Hara, Fin Swanepoel, Christopher H. LeMaster

**Affiliations:** ^1^ The National Collaborative for Restorative Initiatives in Health, Wellington, New Zealand; ^2^ Faculty of Health, Victoria University of Wellington, Wellington, New Zealand; ^3^ Ngāti Ranginui, Waitaha ā Hei, Ngāti Rangiwēwehi, Ngāti Hinerangi, New Zealand; ^4^ Royal Australasian College of Medical Administrators, Hawthorn East, VIC, Australia; ^5^ Ngāti Raukawa Ki Te Tonga, Ngāti Toa Rangatira, Te Arawa, New Zealand; ^6^ ACT Health Directorate, Canberra, ACT, Australia; ^7^ Clinical Excellence Commission, Sydney, NSW, Australia; ^8^ Metro North Hospital and Health Service, Herston, QL, Australia; ^9^ Queensland Health Victim Support Service, Brisbane, QL, Australia; ^10^ School of Nursing, University of British Columbia, Okanagan, Kelowna, BC, Canada; ^11^ Restorative Research and Innovation Lab, Dalhousie University, Hallifax, NS, Canada; ^12^ Community Justice Initiatives Association, Langly, BC, Canada; ^13^ Centre for Restorative Justice, Faculty of Arts and Social Sciences, Simon Fraser University, Burnaby, BC, Canada; ^14^ Healthcare System Safety and Accountability, Winnipeg, MB, Canada; ^15^ Health Systems Innovation Unit, London South Bank University, London, United Kingdom; ^16^ Psychology & Psychotherapy, Behaviour & Developmental Psychiatry, South London and Maudsley NHS Foundation Trust, London, United Kingdom; ^17^ Harmed Patients Alliance, Cambridge, United Kingdom; ^18^ Restorative Justice Council, London, United Kingdom; ^19^ The Healthcare Improvement Studies (THIS) Institute, University of Cambridge, Cambridge, United Kingdom; ^20^ Kaiser Permanente, Oakland, CA, United States

**Keywords:** patient safety, restorative responses, compounded harm, restorative justice, restorative approaches, restorative practice, healthcare harm, adverse events

## Abstract

**Introduction:**

Restorative systems are human centred and distinguished by an emphasis on relational principles and practices. Emerging evidence indicates that a restorative approach holds promise to mitigate and respond to harm in the complex health environment. Advocates are collaborating with clinicians and institutions to develop restorative responses to adverse events.

**Method:**

This paper shares the insights of an international network who have been collaborating to nurture the development of restorative policy and practice in five countries since 2019 (Aotearoa New Zealand, Australia [New South Wales & Queensland]; Canada [British Columbia], England and the United States [California]). Our work is at varying stages of maturity and incorporates co-designing, implementing, and evaluating restorative responses to adverse events.

**Results & discussion:**

The viewpoint provides an overview of the core principles, emerging evidence, and shares our collective reflections about the constraining and enabling factors to development. We recognise that we cannot speak to the breadth of work underway worldwide. Our hope is that by drawing on our experiences, we can offer some thoughts about what a restorative lens offers the future of patient and family involvement in patient safety, whilst providing the opportunity for transparent critique of work to date.

## Introduction

The restorative approach is conceptualised as a broader social movement that holds promise to nurture potentially transformative, more accountable, healing systems that are dynamic and responsive to communities (1, 2). Given the focus on equity and community involvement, perhaps it is unsurprising that advocates are increasingly partnering with clinicians, academics, Indigenous leaders and policy makers to promote the rapid adoption of restorative initiatives intended to mitigate and respond to healthcare harm (conflict, complaints and adverse events). Our vantage point, as an international collaborative, who come together with a shared purpose, provides a broad network and diverse lenses to draw from whilst supporting and promoting development during a period of rapid growth. We recognise that we cannot speak to the breadth of work underway worldwide.

Drawing on our unique and collective experiences of developing restorative initiatives in five countries, (Aotearoa New Zealand [NZ], Australia [New South Wales & Queensland]; Canada [British Columbia], England and the United States [California]) this paper explores the key factors constraining or enabling development. We are mindful that our own experiences are influenced by the interplay between country specific structures, worldviews and cultural norms and that the complex adaptive nature of healthcare delivery presents context specific challenges. To date, our work has been focussed on the application of restorative principles and practices to *proactively* co-design patient safety initiatives and/or *reactively* respond to harm. Implicit is a requirement for institutions to ensure Indigenous practices and the voices of priority populations and those with lived experience are upheld and have a place.

## Background

Two decades ago, the patient safety movement was established with the intention of preventing harm from adverse events. The World Health Organisation defines patient safety as:

“the absence of preventable harm to a patient and reduction of risk of unnecessary harm associated with health care to an acceptable minimum” (3).

The definition is underpinned by ethical biomedical decision-making which aims to balance beneficence (performing an act that benefits someone) and non-maleficence (the obligation not to intentionally inflict harm). The implication is that someone must determine which harm is *preventable* or *unnecessary,* and what risks are *acceptable* (or not) (4). In the aftermath of an adverse event, directly affected individuals - clinicians, patients or families - are usually excluded from these decisions. Context specific medico-legal or safety infrastructure, and its enactment, contributes to subtle or potentially devastating impacts (5). The severity and nature of the harm, and what to do about it, is defined by an investigator enacting specific legal or safety procedures. Using evidence-based approaches to learn and improve system safety is essential, but the quality and efficacy of investigative approaches, especially those which do not involve safety expertise, is critiqued (6, 7). Legal systems also do not reliably produce justice (8). For example, Section 51 of the Evidence Act 1996 in BC Canada affords protections, through legal privilege, to members of committees who investigate adverse events, who cannot be forced to testify, answer questions or produce documents. Whereas, harmed patients and family members are not provided with similar protections, included in reviews, nor are they given access to the committee report (9).

A fundamental premise for restorative justice is that when a ‘conflict’ (e.g., an adverse event) becomes the property of an institution or profession, specific frameworks and practices determine whose voice is credible (10). The resulting response discounts emotion and steals the ability of affected individuals to decide how harm might be addressed, what they need, and who should support their journey. The character of the response is adversarial, dictating how individuals are allowed to interact with each other, what issues they should be engaged with, and who is in charge (11). There is growing evidence that failing to account for the emotionally distressing and potentially traumatising nature of adverse events dehumanises directly affected individuals, and contributes to compounded harm (4, 12, 13). Compounded harm is fast emerging as an urgent public health issue, which has negative impacts on clinicians, patients and families, investigators and the wider community (4, 14). The following definition was developed over a five-year period, using a range of methods, in Aotearoa NZ.

**Compounded harm** emerges from institutional or interpersonal responses to healthcare harm. It is associated with one or more relational or structural violations that inhibit human agency and deny individuals or communities access to the relational resources they need to make sense of, and heal from, a harmful experience in a safe environment (e.g., dignity, mutuality, care); or the structural rights citizens expect (e.g., informed consent, safe healthcare).

When relational or structural rights are violated, compounded harm can evolve and intensify over time, contributing to individual or collective dehumanisation, injustice, interpersonal violence, mental distress, trauma, post-traumatic stress syndrome (PTSS), unjustified blame (of oneself and others), shame, stigmatisation, moral injury, mistrust, inequity, social isolation or suicide. Ultimately, compounded harm can negatively impact how individuals or communities view themselves, or the world around them, eroding their ability to be free of harm in the future

[Wailling (4). p. 237].

Improving mental health is a global priority and requires consideration of how health system processes can promote, erode, or negatively impact well-being (15). The phenomenon is under researched, but emerging work provides insights into the key features, which may be related to country specific frameworks [e.g., (4, 9, 12, 16)]. This paper explores the development of restorative responses as a key strategy to mitigate compounded harm and promote dignity, wellbeing and trust. A restorative response is a set of relational philosophy, principles and practices that can be applied to prevent, mitigate, or respond to healthcare harm and may be used interchangeably with the term restorative approach.

### Restorative principles and practices

Restorative philosophy appreciates that humans are inherently relational beings, and that relationships can heal and harm us (17). A discussion of the rich and diverse roots is beyond the scope of this paper, which attempts to surface key challenges in patient safety, where it has more recently been combined with safety science (11, 18). Different terms are used in our respective countries, often interchangeably, to describe approaches underpinned by restorative principles and goals.

**Figure 1:**
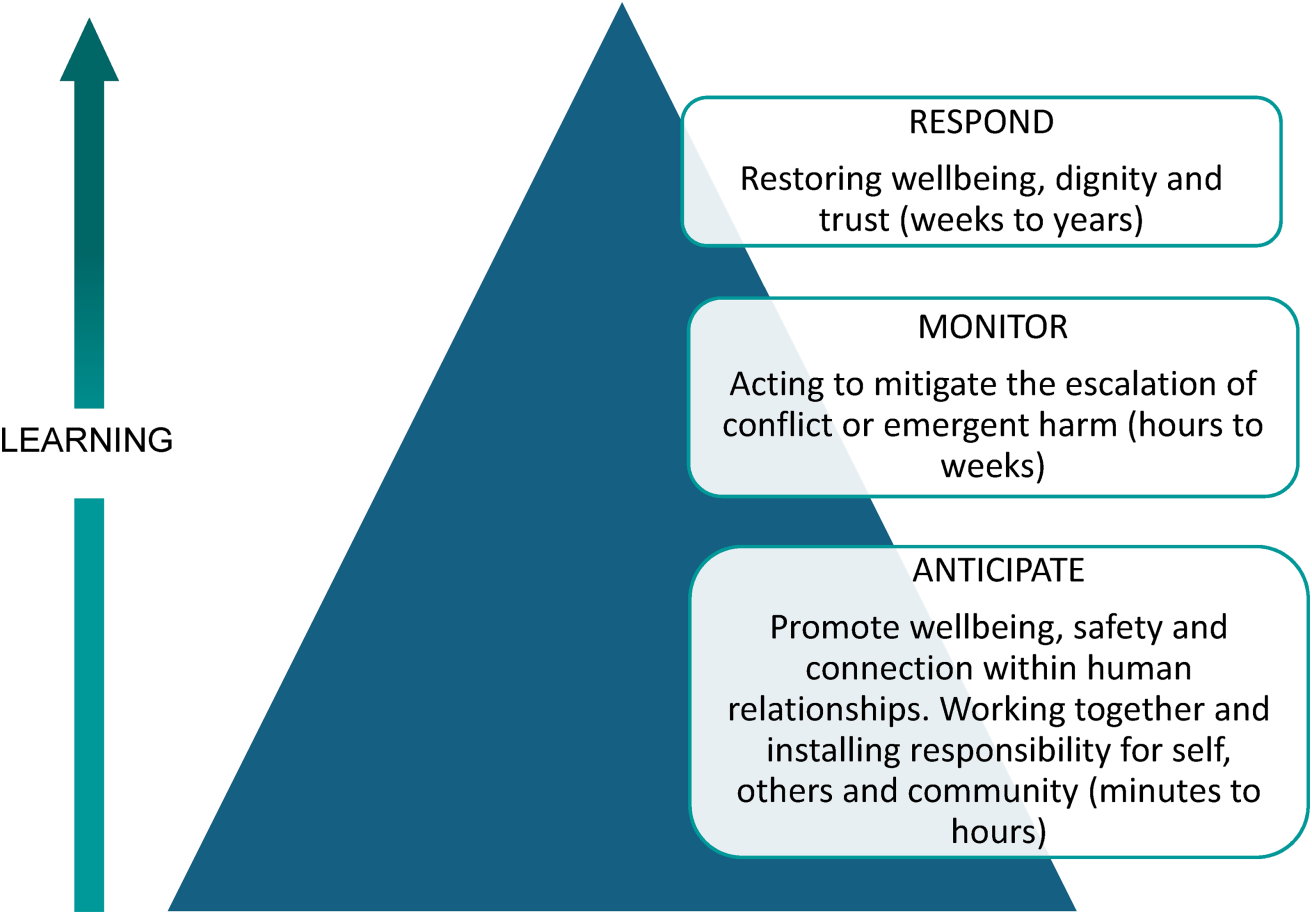
The restorative triangle. Adapted with permission from “Restorative inquiry: A resilient response to systemic harm?” by Jo Wailling, 2021.

A restorative approach may be broadly conceived as a set of relational principles that finds expression in common practices that promote human agency, dignity, respect, voluntariness, responsibility, equity and safety. In the complex health environment, a restorative initiative appreciates the holistic, responsive and dynamic human contribution to safety and wellbeing. The restorative triangle (Figure 1) serves as a visual reminder as to how human relationships contribute, and how relational “slack”^1^ influences system resilience capacity. Ideally, restorative systems focus their efforts on *proactively* promoting safety through strong relationships, anticipating that harm is inevitable in a complex system, and less time in a *reactive* state responding to harm. Developing relational capacity nurtures conditions in which individuals feel more able to have difficult conversations and resolve conflict. Doing so means that when harm inevitably occurs, it is understood as an event worthy of learning and a human experience that creates needs and obligations (4).

A restorative response involves listening to understand what happened, the impacts and needs involved, and clarifying the responsibilities for repair (20). NZ research concludes that the following principles are important considerations in complex health systems (4, 21).

**1. Acknowledge the harm and involve the affected community.** Systemic and individual risks are transparently communicated. When harm occurs, it is approached as an event to be managed and a human experience. The affected community is informed about the potential outcomes and impacts of involvement and can choose to participate in ways that matter to them. Dynamic lived and living experience is validated and responded to.

**2. Respond to the human impacts and needs involved.** Directly affected individuals can access a range of trauma informed supports within (e.g. skilled facilitation, emotional support) or outside of a procedural response (e.g. peer support, psychologist). Directly affected individuals can safely share what happened (or might happen), with the people of their choosing in a manner of their choosing, and their contributions are viewed as a credible source of evidence.

**3. Clarify obligations.** Listen to understand diverse individual and institutional needs associated with healing, learning and improvement and clarify obligations. Honestly communicate if and how risks can be mitigated.

**4. Take responsibility for harm and repair.** Responsibility is taken for: the human impacts (e.g., compensation); systemic issues (e.g., the design of embedded systems); latent conditions; and ensuring actions for repair and risk mitigation are realised. Potential solutions are co-created and account for the diverse perspectives involved.

A common myth is that a restorative response requires a face-to-face meeting, in alignment with the restorative justice conferences used in criminal settings. Whereas healthcare research identifies that offering a range of trauma-informed documentary and dialogical options is valued by harmed patients and families (21). Both can accommodate the use of art, poetry, or other forms of storytelling and stand in contrast to the investigative “interview” or “statement”. Dialogical practices associated with restorative practice encompass a continuum of affective questions and statements, facilitated meetings and Circle practices. Circles involve a structured and intentional conversation in which people, sitting in a circle, respond sequentially to questions posed by the facilitator. Community building Circles create foundations that nurture wellbeing, safety, connection and trust by encouraging collaboration, equitable decision making and cognitive diversity (22).

### Emerging evidence for the use of restorative approaches

In health systems, restorative initiatives have found roots in communities with lived experience of inequity in all its forms and settings where the relational contribution to safety and well-being is incorporated in cultural norms, worldviews, or everyday practices. The key areas of development underway are mental health, women's health, and paediatrics. It is notable that in all of our countries, harmed patients and families, and advocacy organisations are amplifying lived experience voices to inform a community driven approach [e.g., (5, 23)]. In Canada and NZ, initiatives were developed in the aftermath of government inquiries that highlighted health system racism, bias or inequity (24, 25). In these settings, policy development is occurring in partnership with Indigenous communities. In NZ, the sovereignty of Māori over the rights and practices of hohou te rongo (peace-making) as a distinct Indigenous restorative approach is protected by Te Tiriti o Waitangi (The Treaty of Waitangi).

Restorative efforts are focussing on co-designing, implementing or evaluating restorative responses to adverse events or identifying enabling conditions. Table 1 provides an overview of policy and practice initiatives in our countries, and the evidence supporting development. Australian and NZ adverse event policies and restorative guides focus on supporting healing, learning and improvement equitably (42–44). NZ has the most established national approach, being co-designed with a diverse range of stakeholders in the aftermath of a Ministry of Health inquiry (22). As well as providing information about surgical mesh harm and what to do about it, the restorative inquiry identified that compounded harm was widespread and contributed to mental distress, PTSS and suicidal ideation. A descriptive evaluation determined that the restorative response met most participants psychological and procedural needs, captured information crucial for learning, and recommended embedment within systems that mitigate and respond to harm (21).

**Table 1:** Examples of restorative initiatives in our five health systems.

Location	Terms	Practice enablers	Policy enablers	Research/evaluation
Aotearoa New Zealand	Restorative systems, restorative practices, restorative responses, restorative approach. Hohou te rongo (distinct form of peace-making from the Māori worldview)	Ministry of Health restorative response to surgical mesh harm National Collaborative for Restorative Initiatives in Health uses restorative practices to co-create the approach supported by interdependent government agencies. Mental health team capability building sponsored by the Health Quality Safety Commission Māori communities’ (iwi, hapū, whānau) have sovereignty over the distinct but complementary approach of hohou te rongo.	Requirement for restorative responses to be offered in the national adverse events policy, Health and Disability Standards and Mental Health Bill. National restorative health system framework is guiding principles led development Unique legislation supports no fault no blame legislation alongside a Code of Consumer Rights Te Tiriti o Waitangi protects Indigenous knowledge and practice	• A descriptive evaluation of the surgical mesh inquiry concludes that restorative responses should be woven into the embedded system (21) • A realist evaluation of restorative responses in NZ develops a testable definition of compounded harm and eight middle range theories about what works, for whom and in what contexts (4) • A qualitative study concludes that the unique legislation does not mitigate compounded harm for clinicians, advocating for a restorative response to consumer complaints (26). • A study applying social network analysis and qualitative interviews concludes that safety leadership is a responsive relational process (27) • Kaupapa Māori research concludes that adverse event responses contribute to intergenerational trauma and that a culturally responsive practice is required ensuring that each person has their own culture, values and beliefs acknowledged and supported when harm has occurred (28).
Australia (Queensland & New South Wales)	Restorative Just and Learning Culture (RJLC) restorative practice	Queensland state Coroner supports the benefits of using RJLC in coronial matters In NSW, restorative leadership forums raise awareness and build connection Mental health team capability building sponsored by the Clinical Excellence Commission Metro North Mental Health—The Prince Charles Hospital, Queensland uses restorative practices in everyday work	RJLC is incorporated into the NSW safety culture guidance and organisational adverse event policy in Metro North Hospitals Queensland Apology/open disclosure legislation	• A comparative survey and audit evaluation of RJLC initiative concludes improved staff experience, stakeholder involvement and recommendations following suicide (29) • An independent evaluation of a restorative practice model in a secure mental health rehabilitation unit demonstrates efficacy of proactive and reactive approaches (30)
Canada British Columbia	Restorative approach, restorative practice	BC Restorative Circle and Ministry of Health guiding development Restorative leadership forum to raise awareness and build connection Health Quality BC created principles to develop Indigenous patient feedback process	Advocacy to “modernise” a major structural barrier (Section 51 of the Evidence Act)	A scoping review and environmental scan identifies relevant initiatives (8) A co-developed formal research programme includes a feasibility study (31)
England	Learn Together, Restorative justice, restorative just culture, restorative responses, restorative practice, restorative learning	Executive and board commitment in some NHS trusts, and from the Office of the Patient Safety Commissioner Specific organisations advocate context specific approaches to a range of restorative employment and patient safety issues (e.g., Mersey Care, Harmed Patients Alliance, South London & the Maudsley NHS Trust) Restorative Justice Council hosts a Restorative Practitioners in Mental Health Network, with quarterly meetings and annual conference (inaugurated 2016). South London & the Maudsley NHS Trust employ a full-time restorative justice practitioner responsive to patient safety incidents Seven mental health trusts train multi-disciplinary cohorts of staff in restorative conferencing	Learn Together co-developed five principles that encourage restorative learning. The principles inform adverse event policy in participating health organisations and the national Patient Safety Incident Response Framework Royal College of Psychiatry Forensic Mental Health Quality Network Standards for in-patient services include a requirement to enable access to restorative justice for victims, patients and mental health staff (32) South London & the Maudsley NHS Trust made available NHS approved job descriptions to enable the employment of restorative practitioners at three different grades	• Learn Together is founded on a broad programme of participatory research that includes qualitative interviews, ethnography and documentary analysis. It developed guidance relating to how learning responses can incorporate restorative principles to mitigate compounded harm (33). • An independent evaluation of RJLC demonstrated human and economic benefits in a large mental health organisation (34) • A clinical psychology doctoral qualitative evaluation of restorative justice in mental health identifies enablers and inhibitors of up-take of restorative justice (35). • Case series in forensic mental health (36–38)
United States (California)	Not widespread	Unknown Potential to develop into existing Communication and Resolution Programmes	Apology legislation in some states	• A qualitative study involving interviews of 40 patients, family and staff about their experience of CRP programmes recommends that restorative competency is developed (39). • Findings from an exploratory sequential mixed methods study were integrated into a revised version of the “social discipline window” (40) to develop the “restorative accountability’ model which promotes high accountability for institutional citizenship and high support through restorative, non-punitive leadership in academic healthcare institutions (41).

The NZ approach has been informed by Indigenous worldviews and Western research that identifies the relational contribution to safety (see Table 1). To mitigate the risk of marginalising voices or communities, co-design has been shaped within a collaborative framework that is guiding development (44). The expectation to offer restorative responses (restorative practices or hohou te rongo) is embedded within the Adverse Events Policy 2023 and Health and Disability Standards 2022 (42, 45). Section 17(d) of the Mental Health Bill 2024 includes the expectation that hui whaiora (wellbeing meetings) “support restorative practice to uphold the mana (power and authority) of all parties following the use of coercive practices”. Capability building has been underway for two years, initially focussing on workers in mental health settings.

In Australia, mental health has been at the forefront of development, with New South Wales piloting the approach under the umbrella of a *restorative just and learning culture* (RJLC) (43). A development in safety culture thinking, RJLC encourages organisational justice, and ‘forward looking’ accountability, rather than blaming individual clinicians (18). In Queensland, the approach gained traction as part of a Zero Suicide framework at Gold Coast Hospital (46). Their healing, learning, and improving model includes a peer response for clinicians, demonstrated benefits to a range of stakeholders, and enhanced the quality of recommendations (29, 47). A mixed methods evaluation of a restorative practice initiative that balanced proactive and reactive elements in a secure adult community mental health service concluded that the model was beneficial for worker and client relationships. Evaluation participants almost unanimously indicated that there was no downside to introducing restorative practices, identifying that most of the benefits were gained from alignment with the mental health recovery model and everyday use of the proactive elements (30).

The Patient Safety Incident Response Framework in England is based on a programme of participatory research with harmed patients and families that included interviews, ethnography and documentary analysis (12, 14, 48). The ‘Learn Together’ programme incorporates five principles that support a systems based approach to ‘restorative learning’ (49). In British Columbia, a research programme is underway to investigate feasibility and inform contextually relevant, evidence based restorative approaches (50). In the US, an interdisciplinary network is raising awareness about the requirement to enable ‘restorative competency’ within Communication and Resolution Programmes. Academic institutions in the US and Canada have also applied restorative principles and practices to address workplace harms in medical, nursing and dental settings (41, 51).

### What is enabling development?

In our unique and collective journeys, several key factors are enabling the development of restorative potential within our distinctive contexts. Interdisciplinary collaboratives, co-facilitated by individuals with restorative knowledge and skills, have guided development in NZ and BC (44, 52). Indigenous worldview, leadership and voice is ensured and has been integral to advancement. Indigenous communities have many approaches to addressing harm, which can differ by place, be dynamic (shift and change over time), and hold competing perspectives. In the US a similar network connects Western restorative expertise with safety scientists. Opportunities that connect and explore structural and relational interdependencies between institutions, those with lived experience of the system, and communities is essential to build a mutual understanding about what works (or not). Developing relational infrastructure (i.e., collaboratives) is pivotal to ameliorate adversarial relationships and enables the co-creation of systems, key concepts (e.g., safety, harm, justice, responsibility), and supports those involved in the emotional work of change.

Mental health is proving to be a fertile area for policy and practice development and conditions may be conducive for numerous reasons. Firstly, priority populations are overrepresented and there is a strong focus on social justice. Mental health teams work within an interdisciplinary model, have transferable skills, and are familiar with trauma informed dialogical therapies which have some alignment with restorative practices (4, 30). In mental health settings, lived experience is increasingly viewed as a credible form of evidence that has been structuralised into peer worker roles in the UK (53), Australia and NZ. Furthermore, restorative justice is embedded in disciplines used to navigating complex legal and safety matters e.g., Forensics (54). Importantly, most adverse events are suicides, can affect over a hundred people, and thus require a community response (55). Existing suicide postvention services afford a structural opportunity to incorporate restorative principles.

An important first step in a restorative response is to acknowledge that harm has occurred, affects the community providing and receiving care in different ways, and creates unique needs and obligations. Doing so creates opportunities to shape holistic responses and attend to the range of physical, psychosocial, cultural and other needs involved, whilst also learning to improve system safety. It is more challenging to offer a restorative response in the context of adversarial systems, when responses privilege the rights or wishes of institutions or providers, focus on minimising reputational or liability exposure, or are inequitable (9). However, restorative potential can be enabled, and compounded harm mitigated, if approaches explicitly acknowledge and respond to the human experience of harm and participants can make an informed choice about the potential benefits and risks of stepping into procedural responses (4).

Adverse event policy and practice that explicitly acknowledges the complex human experience; enacts an equitable focus on healing, learning and improvement; and expects a range of diverse outcomes is advantageous [e.g., (42–44, 46)]. In Australia and the US, apology protections enable restorative dialogue during open communication and resolution practices (39, 56). An influential institute in England recently proposed that no fault no blame legislation may be advantageous (57). It is important to note that tax payer funded compensation and consumer protections have supported restorative potential in NZ, but can also generate compounded harm if the needs of harmed patients and families are minimised or dismissed (4, 58).

Use of restorative inquiry as a triage tool supports a focus on who is affected and the impacts, needs and obligations involved from the outset of an investigation (29). Furthermore, restorative practices are proving useful tools to enable shared understandings, equitable and safe conditions, honest (often courageous) conversations or the restoration of dignity and trust (21, 30). Early adopters should be aware that restorative responses can result in compounded harm when efficiency is prioritised over quality.

## Discussion

Investment in collaboration and coproduction is a policy enabler for patient safety (59). Restorative responsibility infers broad professional and moral obligations and requires an examination of the voices and contexts that shape how patient safety is defined, responded to, and how system design influences the patient and family experience (4). The rapid adoption or commercialisation of restorative initiatives in healthcare, without a deeper commitment to involving patients and communities might not result in the hoped for and hypothesised change. Marder (60) suggests that the institutionalisation of restorative justice often leads to (re) interpretation, meaning the approach is applied in ways that reflect highly embedded institutional and systemic cultures and practices, that focus on doing things that benefit one party at the expense of another, thus creating inequalities and harms that initially inspired its use. Research examining the prevalence and characteristics of compounded harm, and the human and financial impacts, is essential to raise awareness of the impact of embedded system design.

The term ‘priority population’ reflects a policy and strategy approach that affected communities may reject or view as a label imposed up them by the State. Many of our countries were colonised and the unique harms and needs of Indigenous peoples, and the implications for responsive systems, must be determined by these communities. Western restorative justice can compound intergenerational trauma for Indigenous communities or result in a shift away from its emancipatory and transformative intent (61). Therefore, Indigenous knowledge and practices must be protected rather than being assimilated into Western concepts (62).

To date, RJLC implementation has been overly focussed on institutional goals and supporting clinicians, whilst neglecting to provide the same options to harmed patients and families. An unintended consequence of popularising Wu's ’second victim’ terminology, and excluding voices, is the development of a hierarchy of victimhood that can amplify adversarial conditions [e.g., (63)]. If the goal is to develop restorative potential, these inequities must be addressed, and culturally safe and responsive systems co-designed. Policy that acknowledges patients and families as victim-survivors, rather than a source of evidence, may enable the development of responsive services, ideally independent from investigative matters, and provide access to confidential supports. Evaluation criteria should be co-created with affected communities.

The challenges presented by structural independencies are immense. Culturally safe, trauma informed navigation or support services or harmed patient pathways may offer a way forward (23, 44). In the aftermath of a death, Wailling (4) proposes a further step, in which interdependent institutions collaborate to discharge unique responsibilities within one procedure that is co-designed with the family. Co-design should be used as a tool that distributes power and facilitates cultural responsiveness (64). Research should focus on identifying how responses and their participants might best achieve different ambitions associated with healing, learning and improvement. Given the sociocultural, epistemic and moral issues involved, those concerned with learning to improve system safety may wish to use or extend models that explicitly incorporate these factors [e.g., (65)]; support collective sensemaking [e.g., (66)]; use decolonising methodologies (64); or aggregate and act on the overwhelming amount of recommendations already available [e.g., (7)].

Another potential approach is responsive regulation, which has been utilised in the aged care sector in Australia for some time (67). Responsive regulation is grounded in restorative justice and practice. It involves listening to multiple stakeholders and making a deliberate and responsive choice from a pyramid of regulatory strategies, which are less interventionist and coercive at the bottom of the triangle, and move towards more punitive sanctions (68). Leading safety scholars, suggest responsive regulation may also act as a potential strategy for health system resilience (69). The potential contribution should be explored.

## Conclusion

Restorative approaches are grounded in relational philosophy, principles and practices. Being new to health systems they offer a way to promote wellbeing, dignity and trust and emphasise an equitable focus on healing, learning and improvement. Mitigating the risk of compounded harm from structural and relational violations is essential to promote and maintain human wellbeing. It is possible to enable restorative potential within the context of embedded legislation and policy, but these structures and adversarial practices can constrain development or contribute to compounded harm. Policy makers, practitioners and advocates may wish to invest in development of restorative initiatives in mental, women's and Indigenous health settings which are providing fertile ground to co-design and explore utility. Doing so may also discharge responsibilities to priority populations and requires a human centred collaborative approach that is inclusive of the affected community.

## Data Availability

The original contributions presented in the study are included in the article/Supplementary Material. Further inquiries can be directed to the corresponding author.

## Footnotes

^1^Slack refers to *“available spare resources, of any sort, which can be called on in times of need”* (19).
